# Changing Patterns in Epidemiology of Brucellosis in the South of Iran (2015–2020): Based on Cochrane-Armitage Trend Test

**DOI:** 10.5334/aogh.3474

**Published:** 2022-02-10

**Authors:** Hamed Delam, Zahra Keshtkaran, Behzad Rezaei, Omid Soufi, Mohammad-Rafi Bazrafshan

**Affiliations:** 1 Larestan University of Medical Sciences, Larestan, Iran; 2 Department of Nursing, School of Nursing and Midwifery, Community Based Psychiatric Care Research Center, Shiraz University of medical sciences, Shiraz, Iran; 3 Department of Surgery, Larestan University of Medical Sciences, Larestan, Iran; 4 Shiraz University of Medical Sciences, Shiraz, Iran; 5 Department of Nursing, School of Nursing, Larestan University of Medical Sciences, Larestan, Iran

## Abstract

**Background:**

Brucellosis is known in Iran as an endemic disease; however, its incidence is not the same in all provinces and is higher in some areas.

**Objective:**

The present study was designed to determine the epidemiological status and trend of brucellosis in the period from 2015 to 2020 in the south of Fars province, Southern Iran.

**Methods:**

This is a cross-sectional analytical study. This study included all patients with brucellosis in the period from 2015 to 2020 whose information had been registered in the Infectious Diseases Center of Larestan city. To collect the data, we used a checklist that included information such as age, gender, number of cases by year and month, occupation, and nationality of the patients with brucellosis. The rate of brucellosis incidence during these years was analyzed using the Cochran-Armitage trend test. P-value less than 0.05 was considered significant.

**Results:**

The average annual incidence of the disease was 8.94 per 100 000 population. It has decreased from 26.83 (per 100 000 people) in 2015 to 1.83 (per 100 000 people) in 2020. The results of Cochrane-Armitage process test showed that the reduction in the incidence of the disease from 2015 to 2020 was significant (PTrend = 0.001). The trend of the disease by month also showed that the majority of cases occurred between December and February, with the highest incidence of the disease in February (9.93 per 100 000 people) and the lowest in May (1.1 per 100 000 people) (P = 0.001).

**Conclusion:**

Although the results of the present study indicated a decrease in the incidence of the disease in the southern region of Fars province, due to the fact that the disease is common between humans and livestock, there is the possibility of scattered and irregular epidemics in each period.

## 1. Introduction

Brucellosis, also known as Mediterranean fever, feverish fever, and insane fever, is a bacterial infection that is transmitted from infected cattle, goats, and sheep to humans [1]. This disease is known as Malta fever in humans and brucellosis in livestock [2,3]. The causative agent of the disease is a bacterial species of the genus Brucella [4], and the most important types of Brucella that can cause the disease in humans include Brucella melitensis, Brucella abortus, and Brucella suis [5]. Brucellosis can be transmitted to humans through direct contact with animals or consumption of unpasteurized animal products [6,7]. It can also be transmitted to humans through airborne particles [8]. It can cause localized purulent infections in the spleen, liver, bones, and other parts of the body. It is also known as the thousand-face disease because it has long-term side effects [9]. Symptoms include persistent or regular fever with intermittent periods; profuse sweating, especially at night; weight loss; headache; muscle aches; anorexia; and general body ache [10,11]. The World Health Organization considers brucellosis to be a common human-animal infection that could lead to significant health and economic problems worldwide [5,12]. Brucellosis is widespread worldwide, and it is estimated that there are approximately 500 000 people with brucellosis worldwide, of whom only 4–10% are in developed countries [2,13]. This may be due to vaccine-based control programs [14]. However, brucellosis in low- and middle-income countries is considered a serious health concern [15,16]. Recent reports indicate that brucellosis is endemic or potentially present in 179 countries worldwide and that the disease remains a major health problem in the Mediterranean, the Middle East, West Asia, and parts of Africa, and Latin America [17]. The epidemiology of brucellosis is complex, and Latin American countries such as Mexico and Peru have reported a large number of cases; the same pattern applies to Mediterranean countries such as Iran, the former Soviet Union, Mongolia, and Syria which have the highest annual incidence of human brucellosis [18]. Brucellosis is known in Iran as an endemic disease; however, its incidence is not the same in all provinces and is higher in some areas [19]. Provinces such as Zanjan, Hamedan, Markazi, and East Azerbaijan have the highest incidence and southern provinces have the lowest incidence of the disease [1]. The epidemiology of human brucellosis has changed dramatically in recent decades due to political and socio-economic factors, improved regulatory systems, animal control, tourism programs, and international migration [20]. This disease not only causes physical and health problems, but also imposes economic burden on the society and the government. Fars province and especially the southern regions of the province, because of the livestock and agriculture jobs, are very prosperous; people living in these areas are at high risk for brucellosis. On the other hand, a similar study on the epidemiology of brucellosis in the south of Fars province has not been carried out. Therefore, the present study was designed to determine the epidemiological situation and the trend of brucellosis in the period from 2015 to 2020 in the cities of Larestan, Evaz and Khonj, in the south of Fars province, Southern Iran.

## 2. Method

### 2.1. Type of study

The present study was a cross-sectional analytical research.

### 2.2. Study group

This study included all patients with brucellosis in the period from 2015 to 2020 whose information had been registered in the Infectious Diseases Center of Larestan city.

### 2.3. Study area

**Figure 1:**
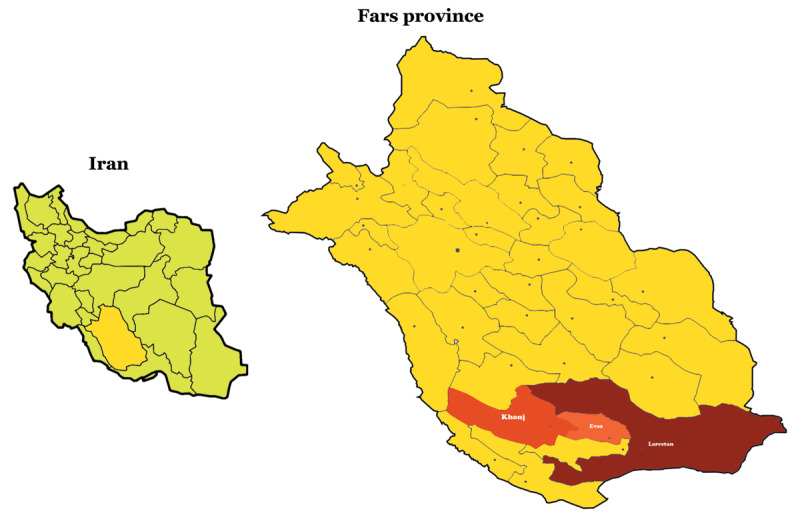
Geographical location of the study.

The study area includes the cities of Larestan, Evaz and Khonj, which are located in the south of Fars province, Southern Iran. According to the latest population statistics registered in the apple system of the Ministry of Health, Treatment and Medical Education of Iran, the covered population in these three cities is about 272 000 people. In terms of climate, Larestan, Evaz and Khonj are considered as arid regions, and the average annual rainfall in these regions from 2003 to 2010 was about 151.8 mm [21]. Larestan city is the largest city in Fars province in terms of area, which is located in the south of this province (***Figure 1***) [22].

### 2.4. Ethical considerations

The present study was the result of research project No. 1399-65 and Code of Ethics IR.LARUMS.REC.1399.013, approved by Larestan University of Medical Sciences. During all stages (completing a checklist and entering data into the software), the patients’ information was kept confidential.

### 2.5. Data collection

To collect the data, we used a checklist that included information such as age, sex, number of cases by year and month, occupation, and nationality of the patients with brucellosis. The researchers investigated all records of brucellosis cases in the Centre for Communicable Diseases of Larestan between 2015 and 2019 from daily visits. This centre records data pertaining to the three cities in the south of Fars province, Larestan, Evaz, and Khonj.

### 2.6. Statistical analysis

After completing the checklists, we entered the data into SPSS, version 25. Descriptive and analytical statistics of the variables are represented by tables and figures. Frequency (percentage) was used to measure qualitative variables. Chi-square test was used to determine the relationship between age, gender, nationality and occupation with the year of the occurrence. The trend of brucellosis incidence during these years was analysed using the Cochran-Armitage trend test. P-value less than 0.05 was considered significant.

## 3. Results

Out of 146 patients with brucellosis, 10 (10.3%) were in the age group of 0 to 15 years and 58 (39.8%) were over 45 years old. Eighty-two patients (56.2%) were male, and the majority of cases were of Iranian nationality (98.0%). Ranchers were the most common occupational groups affected by the disease (64.4%).

The results of Chi-square test showed that there was a statistically significant difference between the age groups and occupations by the year of onset of the disease (P < 0.05), while the variables of gender and nationality of patients during 2015 to 2020 did not show a significant difference (P > 0.05) (***Table 1***).

**Table 1:** Comparison of the frequency (%) of qualitative characteristics of patients with Brucellosis by the year of occurrence.


VARIABLE	TOTAL	2015	2016	2017	2018	2019	2020	P-VALUE

**Age**, years								

0–15	15 (10.3)	4 (26.7)	4 (26.7)	1 (6.7)	1 (6.7)	2 (13.2)	3 (20.0)	0.004

16–30	36 (24.6)	21 (58.3)	3 (8.3)	1 92.8)	3 (8.3)	8 (22.3)	0 (0.0)	

31–45	37 (25.3)	15 (40.5)	3 (8.1)	10 (27.0)	4 (10.8)	5 (13.6)	0 (0.0)	

>45	58 (39.8)	33 (56.9)	6 (10.3)	6 (10.3)	4 (6.9)	7 (12.2)	2 (3.4)	

**Gender**								

Male	82 (56.2)	39 (47.6)	11 (13.4)	7 (8.5)	7 (8.5)	14 (17.1)	4 (4.9)	0.403

Female	64 (43.8)	34 (53.1)	5 (7.8)	11 (17.2)	5 (7.8)	8 (12.5)	1 (1.6)	

**Nationality**								

Iranian	143 (98.0)	72 (50.3)	15 (10.5)	17 (11.9)	12 (8.4)	22 (15.4)	5 (3.5)	0.626

Afghan	3 (2.0)	1 (33.3)	1 (33.3)	1 (33.4)	0 (0.0)	(0.0)	(0.0)	

**Occupation**								

Rancher	94 (64.4)	61 (64.9)	9 (9.6)	9 (9.6)	5 (5.3)	10 (10.6)	0 (0.0)	<0.001

Housewife	17 (11.7)	3 (17.6)	1 (5.9)	6 (35.3)	2 (11.8)	4 (23.5)	1 (5.9)	

Farmer	4 (2.7)	1 (25.0)	1 (25.0)	0 (0.00	0 (0.0)	1 (25.0)	1 (25.0)	

Student	10 (6.8)	4 (40.0)	0 (0.0)	0 (0.00	1 (10.0)	2 (20.0)	3 (30.0)	

Child	4 (2.7)	0 (0.0)	2 (50.0)	1 (25.0)	0 (0.0)	1 (25.0)	0 (0.0)	

Others	17 (11.7)	4 (23.5)	3 (17.7s)	2 (11.8)	4 (23.5)	4 (23.5)	0 (0.0)	


**Figure 2:**
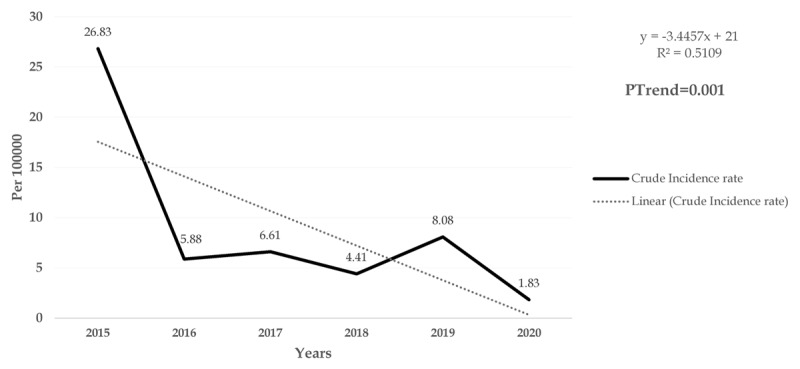
Time trend of Brucellosis incidence by year per 100,000 persons in Southern Fars Province, (2015–2020).

The average annual incidence of the disease was 8.94 per 100 000 population. The incidence of the disease decreased from 26.83 (per 100 000 people) in 2015 to 1.83 (per 100 000 people) in 2020. The results of Cochrane-Armitage process test showed that the reduction in the incidence of the disease from 2015 to 2020 was significant (PTrend = 0.001) (***Figure 2***).

Comparison of the prevalence of the disease by year and season showed that most cases of the disease occurred in winter (46.6%), which was significantly higher than the other seasons (P < 0.001). The lowest prevalence of the disease was in autumn (15.8%). Also, comparison of the prevalence of the disease in each year by the season of occurrence showed that only in 2015, there was a significant difference between different seasons, so that most cases (68.5%) of this disease occurred in winter (P < 0.001) (***Table 2***).

**Table 2:** Frequency (%) of brucellosis cases by year and season in the south of Fars Province, 2015–2021.


YEAR	SPRING	SUMMER	AUTUMN	WINTER	P-VALUE	TOTAL

**2015**	14 (19.2)	2 (2.7)	7 (9.6)	50 (68.5)	<0.001	73 (50.0)

**2016**	4 (25.0)	7 (43.8)	3 (18.8)	2 (12.5)	0.321	16 (11.0)

**2017**	1 (5.6)	4 (22.2)	7 (38.9)	6 (33.3)	0.198	18 (12.3)

**2018**	2 (16.7)	4 (33.3)	3 (25.0)	3 (25.0)	0.881	12 (8.2)

**2019**	4 (18.2)	8 (36.4)	3 (13.6)	7 (31.8)	0.378	22 (15.1)

**2020**	2 (40.0)	3 (60.0)	0 (0.0)	0 (0.0)	0.655	5 (3.4)

**Total**	27 (18.5)	28 (19.2)	23 (15.8)	68 (46.6)	<0.001	146 (100.0)


**Figure 3:**
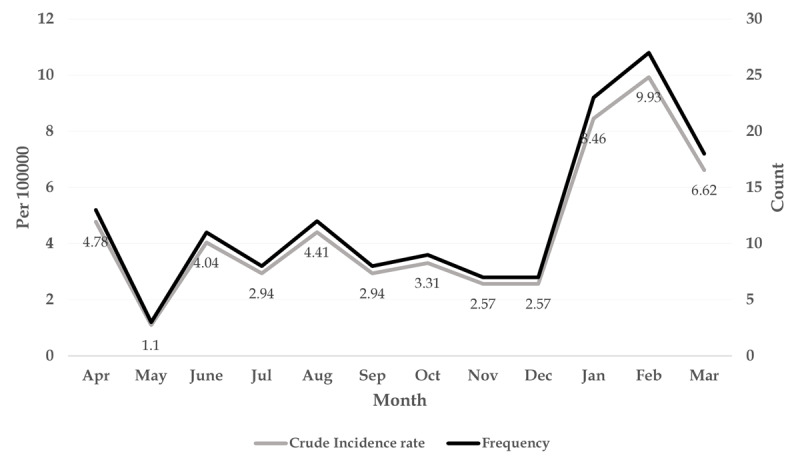
Frequency and incidence of Brucellosis per 100,000 persons per month, south of Fars province, (2015–2020).

The trend of the disease by month also showed that the majority of cases occurred between December and February, with the highest incidence in February (9.93 cases per 100 000 people) and the lowest in May (1.1 cases per 100 000 people) (P = 0.001) (***Figure 3***).

## 4. Discussion

Today, brucellosis is recognized as an important public health issue in many developing countries, especially in the Middle East [23]. In the present study, Cochran-Armitage process test was used to determine the incidence of brucellosis. The Cochrane-Armitage test is used to determine if there are significant changes in the disease incidence during the study period [24]. The results of this test in the present study showed that the average incidence of the disease was about 8.94 per 100 000 population. On the other hand, the incidence of the disease decreased from 26.83 per 100 000 population in 2015 to 1.83 per 100 000 population in 2020, which was also statistically significant. The Ministry of Health, Treatment and Medical Education of Iran presented the classification of Iranian provinces based on the incidence of brucellosis. Fars province is at the moderate level with an annual incidence of 11–20 per 100,000 population [25]. According to the meta-analysis carried out by Mirnejad et al. in Iran, it was found that the incidence of the disease varied from seven (per 100 000 population) in Qom province to 276.41 (per 100 000 population) in Kermanshah province [26]. Another study conducted in Iran during 2011 and 2014 showed that the average annual incidence of brucellosis was about 38.67 cases per 100 000 population [24], which was higher than the incidence rate in the present study; also, another study by Bagheri et al. in 2010 to 2019 in Qazvin province showed that the incidence rate was 27.43 per 100 000 population [27]. However, a study in Greece conducted from 2004 to 2015 found that the average incidence was 1.62 per 100 000 population [28]. In general, there are several intervention and control strategies to reduce the incidence of brucellosis in humans, which can increase the local knowledge about proper food management methods, including pasteurization, extermination of animals infected with brucellosis, and quarantine and isolation [29]. On the other hand, although no vaccine is available to humans for brucellosis, vaccination of livestock is an effective way to reduce the incidence of brucellosis, which is also associated with a reduction in human cases [6]. In the present study, it was shown that the age of the patients was variable and significant from 2015 to 2020, and in general, most patients were over 45 years old. A study in central Iran showed that most cases of the disease were in the age group of 15–20 years [30]. The high incidence of the disease in the adult age group is probably due to overwork and more contact with livestock and livestock products. The results of our study showed that ranchers were the most important and common occupational group related to brucellosis; the incidence of the disease in these people was higher than housewives and other groups. It seems that, due to more exposure to livestock and livestock products, especially when milking, which is done traditionally and manually, ranchers have a higher chance of infection than other occupational groups. Livestock owners usually use raw camel milk to treat haemorrhoids and asthma, animal organs such as the testicles and spleen to treat enuresis, and raw cattle liver to treat anaemia. They also sleep on the skins of newly slaughtered animals to relieve fever and joint pain [31]. In the present study, it was shown that most cases of the disease occurred in winter and the highest incidence of the disease was observed in February. May also had the lowest incidence of the disease. A study in Iran showed that the highest and lowest percentages of the disease were in August and April, respectively [27]. Also, in our study, it was reported that the highest and lowest cases of the disease were in the summer and autumn months, respectively. A similar study in north-western Iran showed that the highest prevalence of the disease was in winter and spring [32]. In this regard, Pakzad et al. showed that the incidence of the disease increased in early spring to mid-summer, and after that it decreased to the lowest incidence in winter; this is not in the same line with the results of the present study [24]. Since the highest rainfall in the tropics occurs from mid-autumn to mid-winter, and, therefore, the amount of forage for livestock consumption during this period increases, the highest amount of milk is produced in this period, which is probably a good justification for the high incidence of brucellosis in winter. Some sources have also shown the role of climate change in the occurrence and further transmission of brucellosis [33].

### 4.1. Study limitations

Because the data collected was obtained through passive surveillance, it may be affected by changes in care protocols such as reporting methods, laboratory diagnostics, and the availability of health facilities over the years. On the other hand, due to the fact that some of the patients’ demographic data were incomplete or missing, it was not possible to analyse such variables.

## 5. Conclusion

The incidence of brucellosis has been declining from 2015 to 2020, with an average incidence of 8.94 per 100 000 population. The highest incidence was observed in February and the lowest in May. Although the results of the present study indicate a decrease in the incidence of the disease in the southern region of Fars province, due to the fact that the disease is common between humans and livestock, there is the possibility of scattered and irregular epidemics in each period. To reduce the incidence of the disease and prevent related problems, it is necessary to carry out strategic plans and control and preventive measures based on the applied management model by health planners. It is also recommended that modelling techniques should be used to diagnose epidemics in the future and detect the changing course of the disease over time.

Educating people, especially at-risk groups such as ranchers, farmers, and nomads, about how to milk and boil it, as well as the proper brewing of meat products, will greatly reduce the incidence of the disease. However, regular vaccination of livestock seems to be the most effective way to prevent and control brucellosis.
